# Player-aware resource compensation in interrupted cricket matches

**DOI:** 10.7717/peerj-cs.917

**Published:** 2022-03-14

**Authors:** Salam Zia, Hannan Bin Liaqat, Hafiz Usman Zia, Xiangjie Kong, Sultan Shamshad

**Affiliations:** 1 Department of Information Technology, University of Gujrat, Gujrat, Pakistan; 2 College of Computer Science and Technology, Zhejiang University of Technology, Hangzhou, China

**Keywords:** OR in sports, Sports, Cricket, Regression, Resource compensation

## Abstract

The International Cricket Council (ICC) uses the Duckworth-Lewis-Stern (DLS) method for resource compensation in interrupted games, which is an upgraded version of the Duckworth-Lewis (D/L) method. In order to compensate resources, the D/L method uses a generic resource table for all teams without considering both teams’ past performance, venue of the match, and players participating in that match. That is why teams cannot strategize according to their strengths and play according to D/L’s requirements. This paper presents a method for Player-aware Resource Compensation in Interrupted Cricket Matches (PRCICM). The PRCICM model is dynamic because it allocates a different number of resources to different teams based on their resource usage in the past, players involved in the match and venue of the match. The proposed method eliminates the need to find the generalized scoring patterns with a player-driven approach. A team-specific approach is more appropriate because of teams’ different formations and playing approaches.

## Introduction

In cricket, a set of 22 players is involved in a game divided into two equal groups of 11. Cricket has different international formats, such as Test Cricket, One Day International (ODI), and Twenty 20 (T20). T10 and 4-day cricket formats are also used in different leagues at the domestic level. A set of six legal balls bowled to a batsman by a bowler is called an over. Bowlers bowl their quota of bowling in terms of overs. A bowler can bowl a maximum of 60 deliveries in an ODI match. This means that one can bowl a maximum of 10 overs. There are multiple ways to dismiss a batman end their inning like run out, bowled, etc. Whenever a batsman gets out, the batting team has lost a wicket. For further and in-depth understanding of the rules of cricket, the below referred web page is a great resource (MCC, 2018). The International Cricket Council (ICC), the governing body of cricket, organizes global cricketing events such as the World Cup, the Champions Trophy, and the International Test Championship. The ICC has also established the ranking systems of players and teams for every game format, and players have been categorized accordingly (Ranking, 2018). The ICC has announced that a league-based structure will be imposed on international tours. There will be separate leagues for all formats, and it will also act as a pathway to qualify for the World Cup (Media, 2018).

The ICC had adopted a system called Duckworth-Lewis (D/L) method for helping them settle matches affected by natural causes such as rain and bad light (ICC, 2018). The D/L method was first introduced in ODIs in January 1997 and is also used in T20s. In the D/L method, when an ongoing match is affected, the algorithm considers the existing scenario and gives its estimation after plotting it on a resource table. If interruptions occur in the first innings of a match, then the remaining overs of the match are reset to a new value to conclude the match within the specified limited time. Also, if rain starts pouring at the end of the first inning or the start of the second inning of a match, then the target score and overs for the second inning are reset for getting match results within the given time constraints. Two scenarios can occur if the interruptions occur in the second inning and the game cannot continue. If the team has played 40% of the overs, then the D/L method can predict the winner of the match; otherwise, the match is abandoned with no result. A similar approach is taken if the match is affected by bad light or any other natural calamity.

In the D/L method, when a match is interrupted in a match’s first innings, the D/L algorithm uses information such as the wickets lost, current score, remaining overs, and the average ODI score. This data is plotted on a resource table to compensate for the lost resources in terms of overs. The interruption in the second innings makes the algorithm choose the first innings score rather than the average ODI score for generating the resource table. That resource table is also used to calculate Net Run Rate (NRR) by analyzing the consumed team resources at the end of a match (Duckworth & Lewis, 2004). The Entertainment and Sports Programming Network has built its model to calculate the impact of each run and wicket on a match. This model also predicts the match’s outcome based on the previous record of players involved in the game (Team, 2019).

D/L methods assign each wicket a resource value and do not care about who the batter is. In cricket terms, the D/L method will assign the same number of resources to Virat Kohli and Chris Martin if they come to bat at the same batting position in a match. Different teams can have different types of batters at different positions. Furthermore, one team may choose to play aggressively at the start and try to finish the game early, while the other may choose to preserve wickets until the end and then play aggressively. That is why D/L’s approach of finding a scoring pattern that gets impacted by all teams rather than a specific team is not the right way to allocate resources.

In order to solve the issues mentioned above, this paper proposes a resource compensation algorithm called Player-aware Resource Compensation in Interrupted Cricket Matches (PRCICM). The proposed algorithm consists of two parts. The first is to find the scoring pattern of individual teams and calculate how much weightage an over has in a team’s inning. The second part is assigning weightage to each player’s wicket based on their career average, host country average, and the average of the last five matches.

In summary, the contribution of the proposed work includes:

• **Different scoring patterns:** PRCICM assigns different over weightage to different teams to help them strategize according to their strength rather than D/L’s generalized percentage.
• **Player’s worth:** Instead of assigning worth to each batting position, PRCICM, using multiple linear regression, allocates worth to each player. That helps accurately measure the most critical and less critical wickets in an inning.
• **Impact of the host country:** Inclusion of average in the host country as a performance indicator for player evaluation makes sure that if a player struggles in any of the countries, then his wicket will have less importance in that specific country.

## Related Work

Cricket is the second most followed sport in the world. It is played between two teams, and the team that scores the most runs is declared the winner (Worldatlas, 2018), *i.e.*, it is a zero-sum sport. When the match is affected by bad weather conditions, D/L is used to compensate for lost resources (Duckworth & Lewis, 1998). It was devised by two English statisticians, Frank Duckworth and Tony Lewis. The paper was initially proposed in 1997, and later on, Professor Steven Stern was added to it, and it was renamed in 2014 to its current title. Initially, cricket used to rely on Average-Run-Rate and Most Productive Overs methods, and these two methods were intrinsically flawed.

In 1992, the World Cup Semi-final was interrupted by rain when South Africa needed 22 runs from 13 balls. The target was revised using the Most Productive Overs method and gave South Africa a target of 21 runs of just one ball, thus robbing them of any chance of making it to the World Cup Final. This game gave Duckworth the much-needed impetus to work on the model (Staff, 2007).

Duckworth-Lewis-Stern (DLS) method is based on resources. Each team has two essential resources, *i.e.*, overs and wickets, to score as many runs to outscore the opponent as cricket is a zero-sum game. The DLS method exploits these available resources’ close correspondence with its final score (Australia, 2006).

### Disadvantages of generalized resource allocation

After introducing the D/L method, criticism started pouring, and a revised method was introduced in 2004 (Clarke & Allsopp, 2001). Even after the perceived improvements, the new method is well criticized by critiques. A survey of the D/L method shows that this method is biased. Studies show that the D/L method favors the team batting first, and teams also use that for gaining an unfair sporting advantage. That study also shed light on how D/L is influenced by wickets rather than runs and run rate (Shah et al., 2015). Another criticism of the D/L method is that the flow of resources is not the same in both innings, and the team batting 2nd can also benefit from that by quickly achieving the D/L par score. In 2009, SE Stern generated separate resource tables differently (Stern, 2009).

Researchers behind the D/L method again defended their mechanism by calling other methods unfair and giving examples of the behavior of the D/L method in some matches with comparison to other proposed methods (Duckworth & Lewis, 2015). Stern also criticized the D/L approach with examples that this method is unfair and the reactive approach of calculating the target is unsuitable. He compared some other proposed methods with D/L and concluded that although D/L is simpler, it still competes with others (Stern, 2016). In the same year, a researcher also proposed a methodology that evaluates the form of teams and home advantage. This method has over by over prediction accuracy of 82% in the 2nd inning (Asif & McHale, 2016). The core issue with D/L is that it starts forecasting after 20 overs. Recently a poster paper claimed that neural networks, Naive Bayes, and Random Forest have better accuracy than the D/L method. Besides optimizing the resource table, they claimed that their model has the same accuracy at ten overs as D/L has at 20 (Abbas & Haider, 2017).

### Influence of T20 on scoring patterns

When T20 cricket arrived, the primary issue with the D/L method was that it was not designed according to the game’s shortest format. Scoring patterns are different in both formats, and batters are less cautious about losing their wicket. A simulator is proposed by researchers for twenty-twenty international (T20I) cricket matches. The good thing about it is that it considers players but does not account for its current form. But for the second innings, these simulation results are dynamic but biased (Davis, Perera & Swartz, 2015). Studies show that the starting of a match has a different approach than the later part. Usually, powerplay overs add more runs to the game than regular overs because of the aggressive nature of opening batters and the applied fielding restrictions (Silva, Manage & Swartz, 2015). McHale and Asif (2013) declared D/L better than all previously proposed methods, but they suggested that the best method to predict the outcome of a cricket match should be the one that works in T20I and ODIs. They proposed a method in 2013 and came up with a better approach in 2016 (McHale & Asif, 2013). Historical data of players and venues has its worth, and by using that, a match can be predicted (Cricviz, 2019).

### Importance of condition and players

Usually, conditions have a significant impact on players’ performances in cricket. That’s why a region-based ranking method is proposed (Daud et al., 2018). Some researchers developed a simulator that takes some historical and instantaneous data points to simulate the game. They also take individual characteristics of batters but leave the bowlers for future work (Sankaranarayanan, Sattar & Lakshmanan, 2014). Statistical algorithms such as Naïve Bayes are also used to predict the match’s outcome with the help of historical data (Singh, Singla & Bhatia, 2015). A team’s performance depends on how the team is formulated, and it also indicates their preferences to win matches. A method has also been proposed to calculate the number of deliveries expected to be bowled in a competition based on the players involved and their strength (Tyagi et al., 2020). In 2017, a method was proposed to calculate the precedence. This can show the strengths and weaknesses of a team and could also be helpful to understand their strategy (Ahmad et al., 2017). Some people have treated player prediction as a classification problem, while others have done the same using regression analysis (Kalpdrum & Niravkumar, 2018; Anik et al., 2018).

## PRCICM Design for Weightage Allocation and Score Calculation

**Figure 1:**
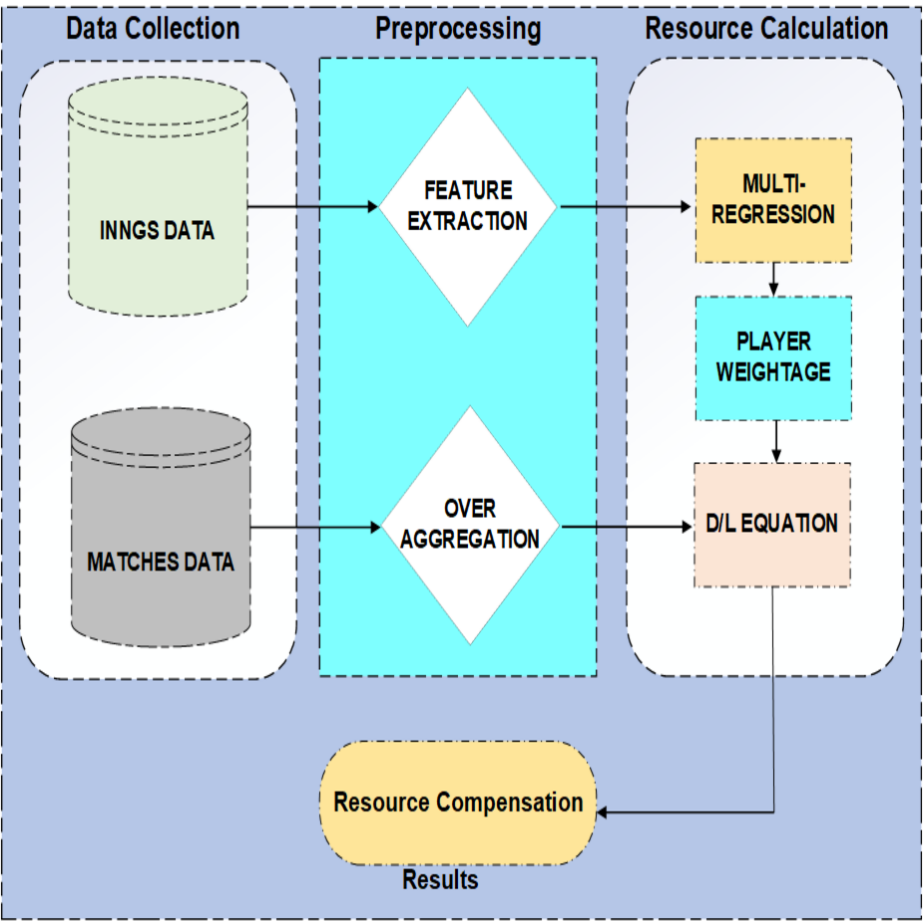
Architecture of PRCICM method.

Figure 1 shows the flow of PRCICM. PRCICM method requires data for two purposes: to evaluate how a team plays in different periods of a match, and the other is to evaluate how much a player’s worth is in his team. Ball by ball data of matches mentioned as matches data in Fig. 1 helps understand the team’s performances, and inning by inning data of players helps evaluate players with respect to other players in playing 11. Inning data does not have some of the required features for this task. That is why career average, average in last five matches and average in host country were extracted.

On the other hand, matches consist of ball-by-ball data, which means that the dataset has multiple samples of a team playing in any period of the match. All those samples were aggregated into a single value for a single over. Multiple linear regression determines the expected performance of a player, and that score then results in the weightage of each player. Multiple linear regression was applied on inning data, and that data was divided by the host country. Player weightage along with aggregated over data results in how much a team is worth in any given scenario, and that worth, with the help of Duckworth and Lewis’s proposed equations, gives the final result.

The following data sets are used for this research:

i. Inning by inning data of 148 players from top 10 ODI teams
ii. Ball by ball data of all ODI matches from 01/01/2006 to 14/12/2019

The dataset (i) was taken from Kaggle. Ball by ball data numbered as (ii) was downloaded from https://cricsheet.org/.

### Over aggregation

Individually for all teams, each over of the innings was assigned a value or worth by the following formula:

(1) \begin{eqnarray*}{\mathrm{O}}_{\mathrm{W}}= \frac{\text{Runs Scored in that over}}{\text{Total Runs Scored}:} \times \mathrm{C}\end{eqnarray*}

In Eq. (1) C is a constant, and its value is 50. This value indicates that deliveries and wickets have an equal share in total resources at the beginning of the inning. The assumption that led to Eq. (1) was that each team could have a different resource usage strategy, giving them the freedom to change or continue their playing style.

**Figure 2:**
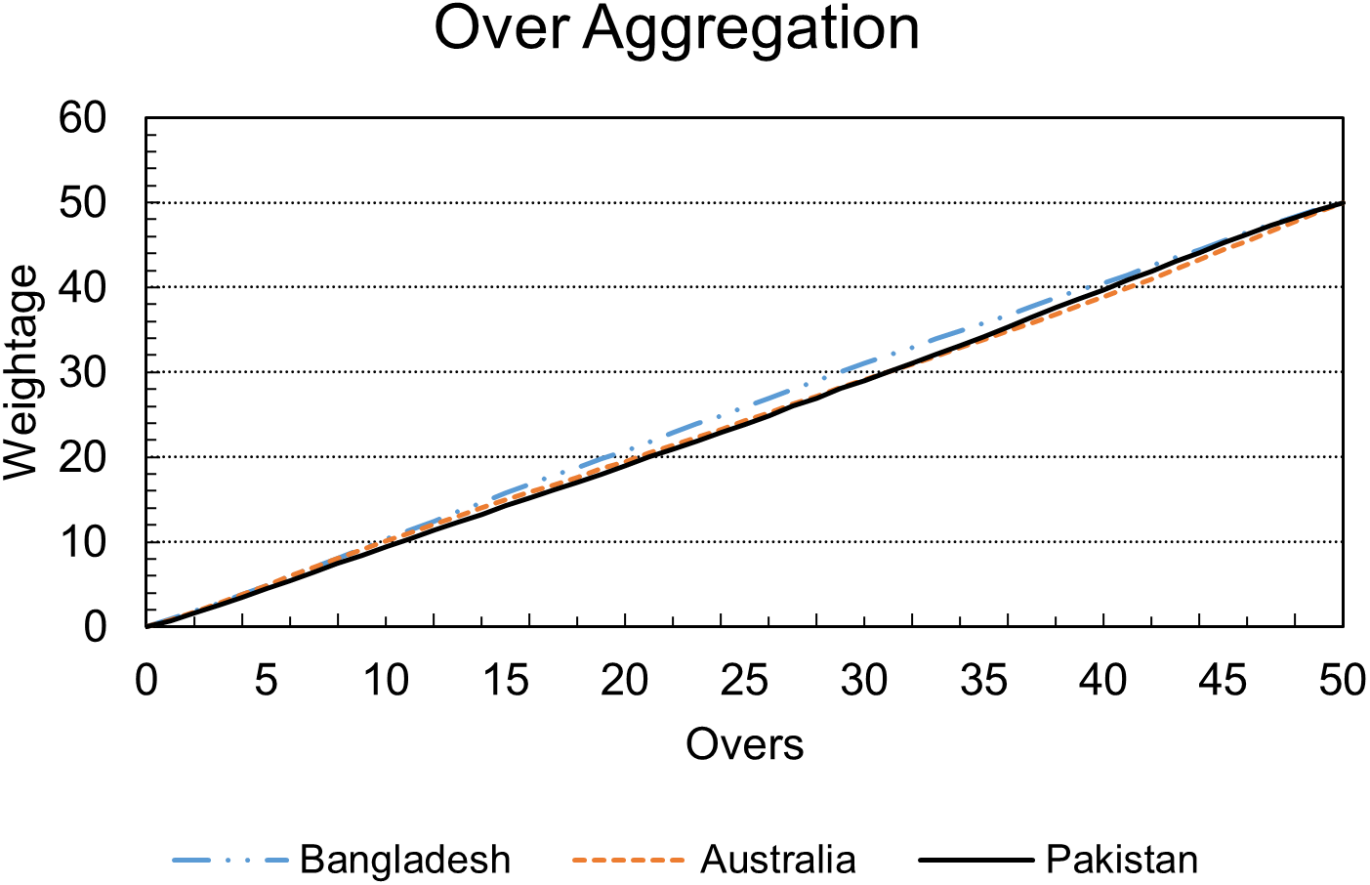
Accumulated over by over weightage.

Figure 2 validates the assumption mentioned above as it can be seen that Bangladesh has a very different trajectory than Australia and Pakistan. Also, it can be seen that initial overs contribute less to Pakistan’s inning, and Pakistan’s over weightage is almost similar to Australia. Still, in the last part of the inning, Pakistan’s trajectory is more inclined toward Bangladesh’s scoring pattern.

### Player weightage allocation

The weightage of a player indicates how much that player will contribute to the team’s total score in the next match in the form of a percentage. The Player Weightage Allocation is a way to identify the importance of a player in his team. Dataset(i) is the source of this experiment, and it helped in calculating the weightage of a player using past performances. One crucial factor involved in this process is calculating the weightage of players based on playing conditions. In order to measure the condition-wise impact, a list of 10 countries was created. That list includes Pakistan, India, Sri Lanka, Australia, England, South Africa, New Zealand, West Indies, UAE, and Bangladesh. Nine of the chosen countries were selected based on the official ODI ranking. Since there were no matches played in Afghanistan, Afghanistan is not part of the list. In the recent past, UAE has hosted a significant number of matches. That’s why the UAE was also included in the list.

Only innings played in that particular country were considered for each country in the list. Since cricket teams also have bowlers and they don’t have batting as their core skill, there was a possibility that there could be bias if all the innings of a batter were in the training dataset while all the data of a bowler was included in the testing dataset. In order to remove this bias, each player’s innings data was separated into two parts. One part consists of 80% of the innings, while the other has 20% of his inning data. These separate datasets of all players were combined to form two complete datasets for training and testing. If a player had played only one inning in any country, in that case, he was not considered a part of any of both datasets.

At the time of model selection, there are several options to consider. One thing that impacted the model selection decision is that the dataset is divided into ten parts. Each part must be evaluated on its accuracy and will have no impact on others. From the accuracy and prediction point of view, it can be a linear or non-linear model, but after some analysis, we found out that linear regression is working well with our data, and having different algorithms for different countries might not be a good approach because of different calculation criteria. Previously, in Net Run Rate and team or player ranking calculation, International Cricket Council didn’t use different criteria based on playing condition or country. D/L also maintains that in their approach and PRCICM is also based on that approach.

In order to check multicollinearity, VIF was used on each country’s dataset separately, and the score for each parameter in their respective dataset is presented in Table 1. According to (O’brien, 2007), this score is satisfactory, and there is no need to reevaluate the independent variables.

**Table 1:** VIF of features for each country.

Country	Overall	Last 5 Matches	Host Country
Pakistan	6.573117	7.158426	2.082441
India	6.507897	3.712919	4.912342
Sri Lanka	5.637875	4.060742	3.683128
Australia	6.199958	4.775388	3.597006
England	5.498104	4.724606	3.408363
South Africa	6.898698	5.016591	4.606784
New Zealand	7.030213	4.894000	3.601747
West Indies	6.277596	3.981354	3.475201
UAE	5.734885	4.236799	3.322601
Bangladesh	6.020243	4.792490	3.905624

The training dataset of each country was then passed to multiple linear regression separately to find out the weightage of each parameter in the dataset. Runs were used as the dependent variable, while career average, average of last five matches, and average in the host country were independent variables. Since each country has separate datasets for training and testing, multiple linear regression generates separate equations for every country. The generated equation for each country is given below in Table 2.

**Table 2:** Country wise weightage of parameters.

Country	Equation
Pakistan	Runs = 0.492847 * Overall −0.19427 * Last 5 Matches + 0.115698 * Host Country + 17.32622
India	Runs = 0.38272 * Overall −0.03755 * Last 5 Matches + 0.218038 * Host Country + 12.15356
Sri Lanka	Runs = 0.6636162 * Overall + 0.01683079 * Last 5 Matches − 0.05041108 * Host Country + 6.261548
Australia	Runs = 0.5222344 * Overall + 0.05455908 * Last 5 Matches + 0.06997351* Host Country + 8.619372
England	Runs = 0.6795543 * Overall − 0.05491578 * Last 5 Matches + 0.08635106 * Host Country + 7.504847
South Africa	Runs = 0.4389146 * Overall + 0.08165265 * Last 5 Matches − 0.00648676 * Host Country + 11.98543
New Zealand	Runs = 0.6025601 * Overall − 0.0251784 * Last 5 Matches + 0.00478968 * Host Country + 10.10386
West Indies	Runs = 0.6103091 * Overall − 0.01049663 * Last 5 Matches − 0.00174947 * Host Country + 9.342105
UAE	Runs = 0.3208915 * Overall + 0.01092727 * Last 5 Matches + 0.17579547 * Host Country + 12.13873
Bangladesh	0.5355541 * Overall + 0.08445139 * Last 5 Matches + 0.12409565 * Host Country + 5.17619

This equation can predict the score of any player in any of the countries mentioned in Table 2 with the help of the player’s stats. If a player is on his international debut, then the player’s record that he is replacing will be used as his stats. Some of the variables negatively relate to the score in some countries. For instance, the average of the last five matches in Pakistan has the strongest negative relationship compared to other negative values, and West Indies has two negatively impacting parameters. There could be a slight chance that the predicted score of a player turns out to be a negative value if any of the players have a bigger value of the negatively impacting parameter. Although none of the predicted values was negative during testing, to remove this exception, the absolute value of the prediction was taken. It will have no impact on normal predictions but assign the negative value as a non-negative to the player.

The predicted score of each player in a team was used to calculate G50. G50 represents the total expected score of a team if they utilize all their resources. A team in cricket has 11 players, so G50 for a team will be the sum of all 11 players playing on any given day. Suppose S is the predicted score of each player in a team, then for each predicted score, the equation below shows the formula to calculate G50.

(2) \begin{eqnarray*}\mathrm{G}50=\sum _{\mathrm{K}=1}^{11}{|}{\mathrm{S}}_{\mathrm{ K}}{|}\end{eqnarray*}

In order to calculate the weightage of each player, his expected score is divided by G50. Suppose W is the weightage of each player then for each player P in the playing eleven:

(3) \begin{eqnarray*}{\mathrm{W}}_{\mathrm{P}}={\mathrm{S}}_{\mathrm{P}}\mathrm{ \ast D}/\mathrm{G}50\end{eqnarray*}

In Eq. (3) D is a constant, and its value is 50. This weightage was combined with over’s worth, already stored in the dataset (ii), then used in the PRCICM method to determine how many resources a team has before and after an interruption.

### Resource compensation

PRCICM method is a resource compensation algorithm that takes values of lost resources and then assigns a score that the team under consideration gets for those resources. PRCICM method changes the underlying working of the DLS method and uses a different method to assign resources to each wicket and over. Instead of using a one-size-fits-all approach, the PRCICM method treats each team and player separately and uses a generalized but more dynamic approach than DLS.

At the beginning of a match, each team has 100% resources. Resources were divided into two parts one is for players, and the other is for overs. When either ends, total resources became 0 as the team has either no batter to send in for batting or they have no over available to bat. The worth of each over and every player is calculated using Eqs. (1) and (3), respectively, so at the beginning of any match total worth that any of both teams involved in a match can be calculated using the equation below:

(4) \begin{eqnarray*}R=\sum {O}_{W}+\sum {W}_{P}\end{eqnarray*}

D/L denotes R_1_ as the resources of the team batting first, and R_2_ represents the resources of the team batting 2nd. If an over has bowled in a match, then the worth of that over will be subtracted from R. Similarly, if any player has lost his wicket, in that case, the worth of that player will also be subtracted from the total resources of the team.

As the match progresses and a team starts consuming overs, their total resources will also reduce, as described above, but what if they don’t lose any wickets even until the 49th over of a match. In order to cope with this anomaly, another formula is proposed that only focuses on the total remaining worth of players and reduces their total worth accordingly. Suppose P_RES_ is the total worth of all not-out players in a team’s inning at the interruption, and O_B_ is the total number of overs that the team has faced before the interruption, while C is the total allocated overs to that inning.

(5) \begin{eqnarray*}{P}_{LOSS}={P}_{RES}\ast {O}_{B}/C\end{eqnarray*}

Equation (5) helps reduce resources so that even if a team has ten wickets in hand at the interruption, the total consumable number of resources represents the extent to which the players in the team can take the target rather than just relying on their possible potential. That is the reason to subtract the value of P_LOSS_ from R.

PRCICM method allocates total resources to a team using Eq. (4). It then subtracts the value of consumed overs, the worth of each player that has lost their wicket, and P_LOSS_ from R. This subtraction returns the total resources that the team still has. In order to calculate how many resources a team has been deprived of, the value of R is calculated before and after interruption and then passed on to the proposed equations of D/L. D/L has two equations for two conditions based on resources. If R_2_ ≤ R_1,_ then:

(6) \begin{eqnarray*}\mathrm{T}=\mathrm{S}{\mathrm{R}}_{2}/{\mathrm{R}}_{1}\end{eqnarray*}

In the case of R_2_ ≥ R_1,_ the proposed equation is:

(7) \begin{eqnarray*}\mathrm{T}=\mathrm{S}+\mathrm{G}50({\mathrm{R}}_{2}-{\mathrm{R}}_{1})\end{eqnarray*}

In Eqs. (6) and (7), T denotes target while R_1_ and R_2_ are team one and two resources, respectively. D/L uses the average ODI total as G_50,_ but the PRCICM method has a different way to calculate this value, as described in Eq. (2). With these equations, predefined rules to set the value of T were also used in the PRCICM method. Pseudocode for resource calculation is given below:

**Table:** 

**Pseudocode to calculate remaining resources using PRCICM.**	
Initialization	
1.	*reg*←*get host country’s equation from* Table 2
2.	*list*←*list of all players in team*
3.	*diction*← dictionary *(player, score)*
4.	oConsumed ←*Sum of consumed over’s worth from dataset (ii)*
5.	**forall** player *p* in list **do**
6.	*data*←*dataset (i)*
7.	*pavg*← current career average of *p*
8.	*lfm*← average runs scored in last five matches by *p*
9.	*cavg*← average in host country by *p*
10.	*diction[p]*← absolute (*reg.perdict* (*pavg,lfm,cavg*))
11.	**end for**
12.	*G50*←*diction.sum* ()
13.	D ← 50
14.	**for all** score s in diction **do**
15.	*W*_*P*_←diction[s]*D/G50
16	**end for**
17.	*outlist*←*list of all players who got out*
18.	*P*_*RES*_← 50
19.	**for all** player p in outlist **do**
20.	*P*_*RES*_←*P*_*RES*_ - W_P_
21.	**end for**
22.	Calculate *P*_*LOSS*_ Using Eq. (5)
23.	*P*_*RES*_←*P*_*RES*_ - *P*_*LOSS*_
24.	*O*_*RES*_← 50
25.	*O*_*RES*_←*O*_*RES*_ - oConsumed
26.	R ←*O*_*RES*_ + *P*_*RES*_


## Performance Evaluation

In this section, we evaluated the performance of the proposed PRCICM method and compared the results with DLS’s results in actual cricket matches. Other performance indicators were also discussed to differentiate between both methods.

### Evaluation of allocated resources to players

Multiple linear regression assigned weightage to the parameters under consideration. Since the dataset is divided into the country-wise inning, every country has its weightage for every parameter, and their accuracy was checked separately. Kalpdrum & Niravkumar (2018) predicts scores using classification and sets class boundaries with a difference of 24 runs. That is why the accuracy of the proposed method in this paper is measured using that similar criterion.

**Figure 3:**
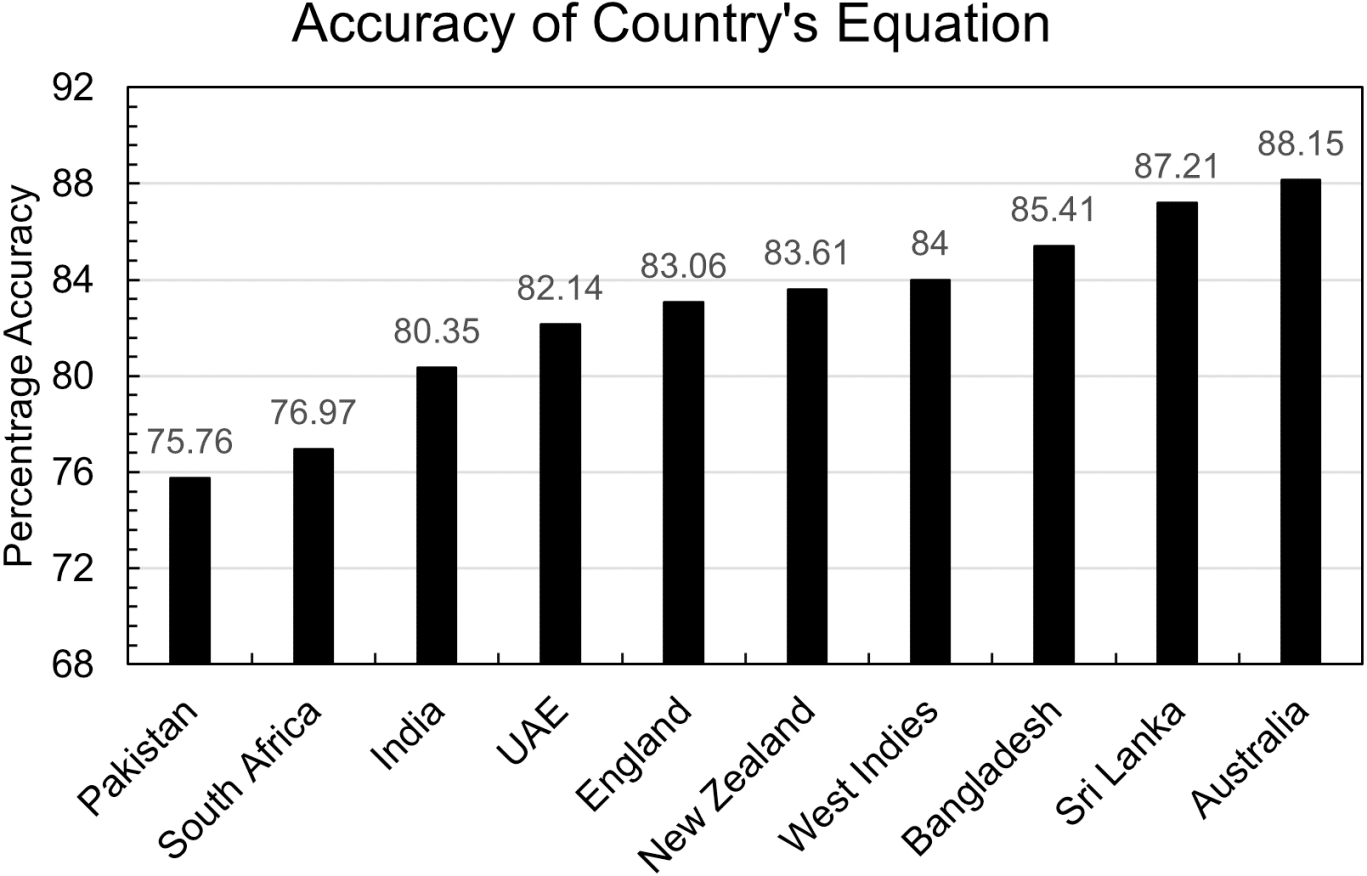
Accuracy of country’s equation.

A total of 1,896 predictions were made on the testing dataset of each country using the equation of that country mentioned in Table 2. The accuracy of each country’s equation is calculated as shown in Fig. 3.

Every country has accuracy greater than 80% except Pakistan and South Africa. One benefit of the doubt in Pakistan’s case that can be given to the model is that the dataset is small, but that can’t be the case in South Africa’s evaluation. So further investigation indicates that the countries in which batters score 50 or more runs more frequently have less accuracy than others. This is also true for the opposite assumption: countries where batters score 50 or more runs less frequently have more accuracy than others.

**Figure 4:**
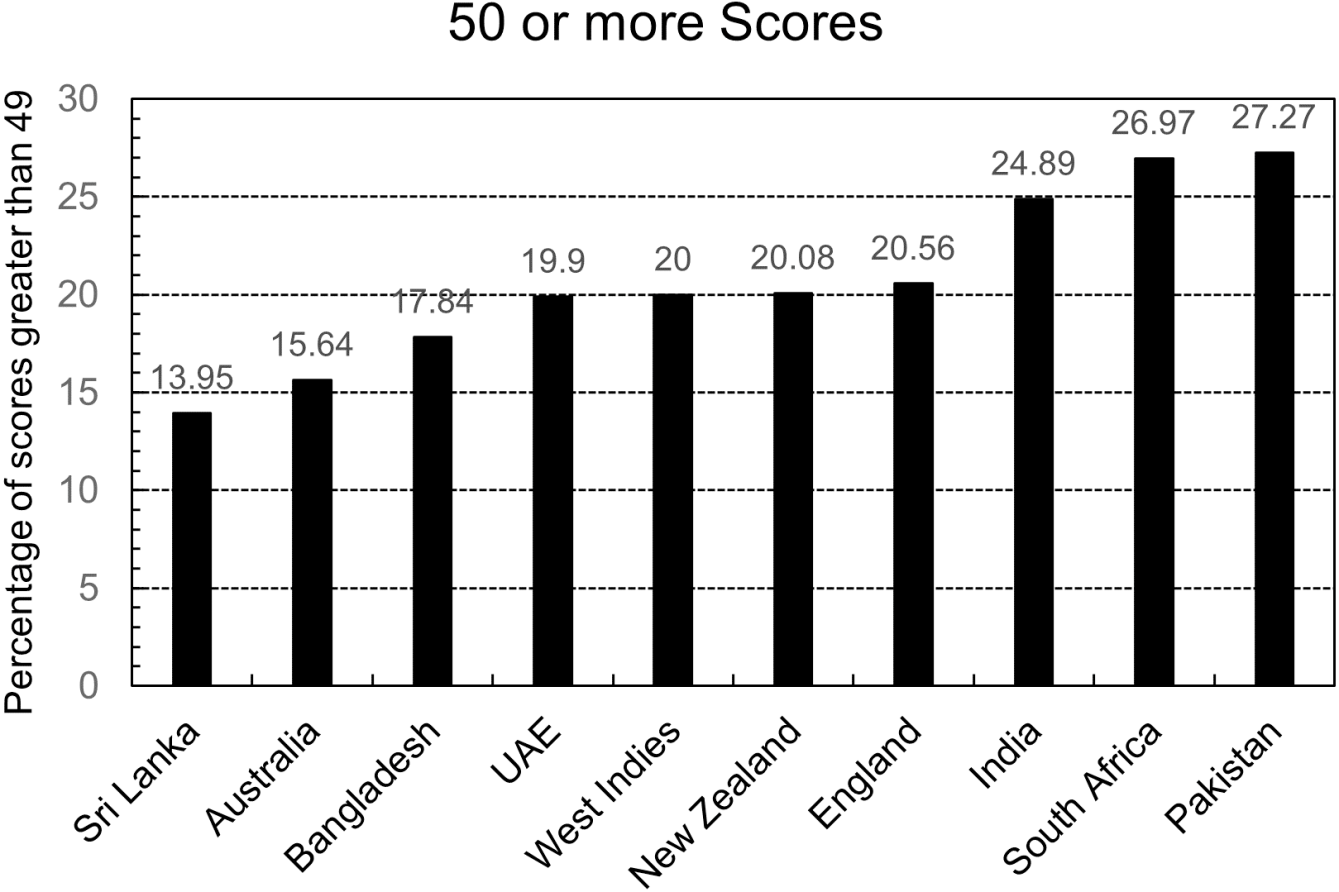
Country wise score comparison.

Figure 4 shows the percentage of scores greater than 49 in the testing dataset of each country. It is evident that the three countries, Australia, Sri Lanka, and Bangladesh have the most accuracy but have the least percentage of scores greater than 49. In comparison, the three countries with the worst accuracy, Pakistan, India, and South Africa, have the most percentage of 49 plus scores in their respective countries.

**Figure 5:**
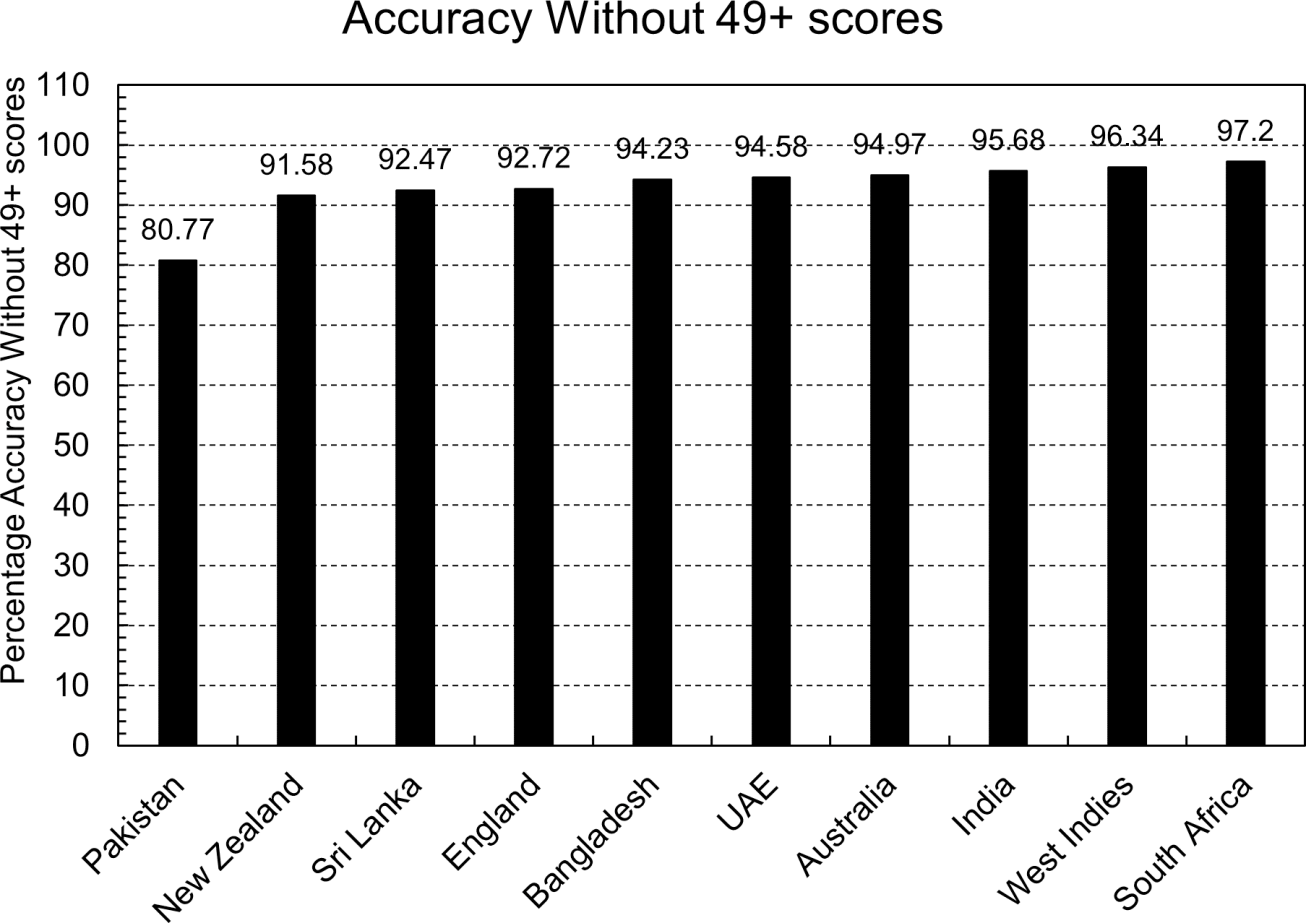
Accuracy of country’s equation without 50 or more scores.

This argument gets further validation with another experiment. If the above-written arguments were correct, then there should be an increase in the accuracy of each country’s predictions when there are no scores greater than 49 in the testing and training dataset. This experiment was performed, and Fig. 5 shows the accuracy of the equations. The accuracy of each country’s prediction has increased, but South Africa and India’s accuracy has increased the most. For both these countries, the correct predictions of the models were almost the same in both scenarios. Still, the number of wrong predictions saw a significant drop, resulting in more accuracy.

Predictions are required in a match’s context, and the predicted score is the criteria to assign weightage and calculate G50. That is why the model is also evaluated from a match’s perspective. A maximum of 11 players can be part of a team, so total predictions were divided by 11 to get the total number of matches, and then the correct number of predictions was divided by that number. That can be interpreted as the expected number of correct predictions in a match.

**Figure 6:**
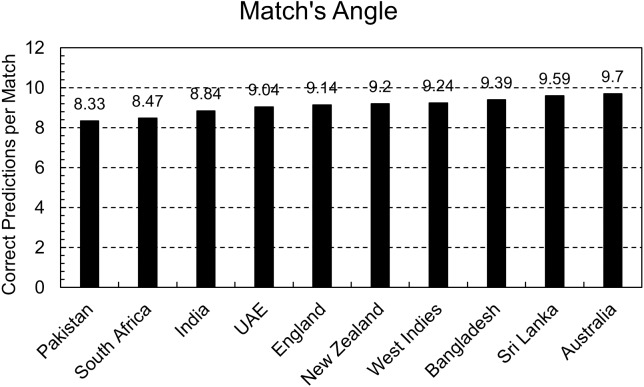
Correct predictions per match.

Figure 6 shows that every country has nine correct predictions per match except for Pakistan, India, and South Africa, and 2–3 predictions can go wrong in a match. As addressed above, those players who perform better than expectations are usually not so accurately predicted. That means a player with higher ability gets lower weightage than expected. These assigned weights are calculated to use in the PRCICM method, and the PRCICM method deducts the full weightage of a player from the team’s resources when he gets out. Thus, those wrong predictions can’t significantly impact the PRCICM method.

### Analysis of PRCICM method in comparison with DLS

**Figure 7:**
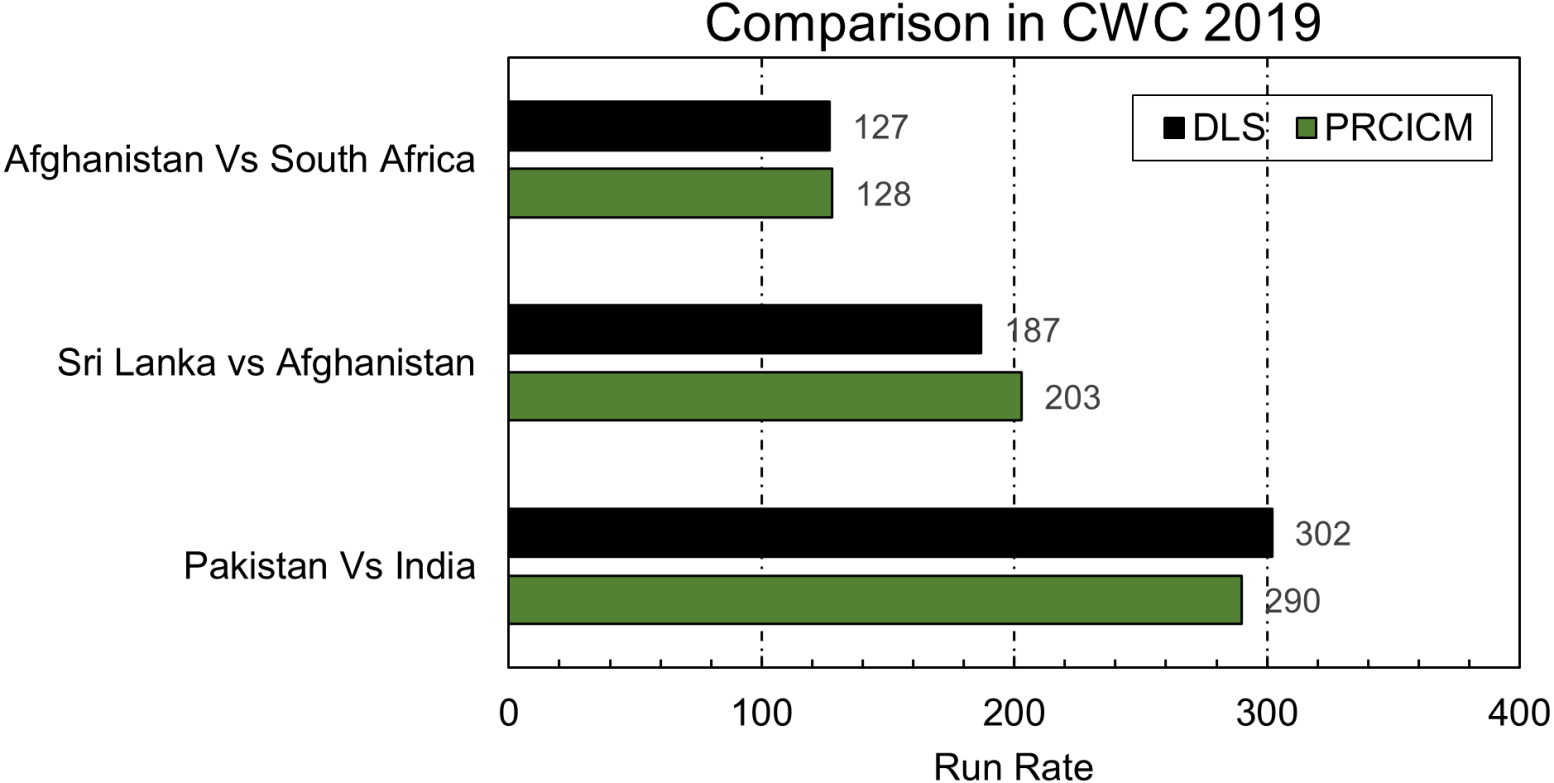
Comparison of PRCICM and DLS in CWC 2019.

ICC uses DLS for resetting targets in interrupted matches. Although the creators of D/L and some others have suggested it for calculating victory points that can be an alternate for NRR, its primary job is to adjust targets based on available and lost resources in a match. PRCICM method is a proposed alternative to the DLS method. With the help of the player’s weightage and over’s worth PRCICM method was applied to all the matches in the cricket world cup 2019 where ICC used the DLS method. Figure 7 shows a comparison of the PRCICM method with the DLS method.

PRCICM method uses players as resources, so every player’s data in playing 11 of both teams must be available. Some players are not available in the dataset, but they were part of actual teams in the aforementioned matches. That’s why their replacement players were used using information from their respective team’s world cup squad. In Afghanistan *vs.* South Africa, Muhammad Shahzad was considered a part of the Afghanistan team instead of Ikram Alikhil, and Dale Steyn’s stats were used instead of Beuran Hendricks. Suranga Lakmal was the missing player from Sri Lanka *vs.* Afghanistan match in the dataset, so Jeffrey Vandersay was his replacement for testing purposes. In Pakistan *vs.* India’s match, Shaheen Afridi and Junaid Khan replaced Wahab Riaz and Muhammad Amir.

In the first match, the match was reduced to 48 overs because of rain interruption. Rain interrupted at 20th over of Afghanistan’s inning, so both methods evaluated the lost resources similarly, and the difference between PRCICM’s target score and DLS’s target is only one run. In this kind of scenario where interruption occurs in the 1st inning, both the DLS and PRCICM method considers the lost resources of the 2nd team because they also have fewer overs to play because of interruption.

The interruption scenario of 2nd match, Sri Lanka *vs.* Afghanistan, is similar to this. However, the DLS method weighs a bit more in favor of Afghanistan’s loss of 9 overs and reduced Sri Lanka’s target, but the PRCICM method’s resource worth is different than DLS, increasing Sri Lanka’s target. This result is debatable, and based on their interpretation of the game, experts can interpret this differently based on their cricketing instincts. The third match was between Pakistan and India. According to the PRCICM method’s evaluation, Pakistan has lost more resources because of interruption, but DLS assigns a lesser number to lost resources in this case. That is why there is a difference between both models’ allocated scores. That shows how the PRCICM method works typically in these scenarios and does not produce any result that can be deemed an odd result. Although PRCICM uses D/L’s equation, resource allocation of both methods is different, and PRCICM has separate over weightage for each country and uses a very dynamic player weightage allocation method. That gives the PRCICM method the ability to evaluate the team’s resources based on their inning trajectory in the past combined with players’ weightage based on their past performances and the country they are playing in.

In order to get good results from the DLS method, the team has to plan their inning according to DLS’s generalized resource table that is used for every team, but the PRCICM method has the resources generalized for every team separately. That is why who got out impacts the PRCICM method, and it caters to changes in the batting order, but DLS does not bother that. In the scenario of Sri Lanka *vs.* Zimbabwe match, mentioned by the creators of DLS, when applied to the Indian team that played the group match *vs.* Pakistan in cricket world cup 2019 and instead of sending Kohli in, Chahal bats at number 3. That impacted the final target by four runs.

As investigated by stern D/L under estimated lower-order partnerships, the match stern mentioned is checked using the same Indian team mentioned above in English conditions. PRCICM method suggests that the Indian lower order did not have what stern expected from the lower order. That is why the PRCICM method produces a result more inclined toward D/L than DLS in that scenario.

PRCICM method failed in one extreme case scenario defined by D/L that if team A and B hit six runs off every ball and then interruption happens at the end of 25th over of 2nd inning, the realistic target is 901 runs. PRCICM methods assigned a target of 904 runs to the Indian team used in previous experiments. From D/L’s perspective, this is a failure because they think if both teams are equal all the time, then, in the end, both should have been equal, but PRCICM is a prediction-based model, and in this scenario, the prediction was India is three runs short.

The famous 2003 world cup final that justifies the D/L method was also passed as a test case to the PRCICM method. India was losing the match by 20 runs according to D/L, but the PRCICM method increased this margin to 66 runs. In this case, the PRCICM method is by far closer to reality.

## Conclusion

In this paper, we have proposed PRCICM, an algorithm to compensate resources in interrupted matches. In contrast to D/L’s one size fits all approach, PRCICM assigns weightage to each player and team separately, resulting in resource compensation closer to reality. Over by over weightage assignment helps find scoring patterns for teams, and multiple linear regression is used to calculate the expected impact of players. In the case of the PRCICM method, teams can develop strategies without worrying about batting order because it is aware of players involved in a match and detects the changing dependency on any player in a team. PRCICM can serve the game better as it understands the capability of players involved in the match, and it will assign them resources accordingly. This resource allocation will produce results closer to reality and bring more fairness to the game. The disadvantage of PRCICM is that it needs player-specific data too. This means that if a player is new to the game, PRCICM will not have sufficient data about that particular player, and that could result in wrong resource allocation. PRCICM is practically adaptable and can be used by ICC for compensating resources. There is no obstacle in implementing PRCICM.

In the future, we plan to propose a more detailed 2nd inning specific approach to perform resource compensation for teams and also for the worth calculation of players and overs. That will help understand the difference in scoring patterns between a team batting first and second. Furthermore, PRCICM needs a detailed domestic to international performance conversion analysis to remove the need for replacement players.

## Supplemental Information

10.7717/peerj-cs.917/supp-1 Supplemental Information 1 The inning data for training the model and the code file that calculates remaining resources (1). *woa.csv* consists of batter’s inning by inning data for training the model. (2). *list.csv* contains the same data as defined in *woa.csv*. This information required to calculate the current career stats of batter. (3). ***bats.sql*** has tables and each table represents one country with pre-allocated weightage to every over of that country’s inning. (4). *predict.py* is the code file that calculates remaining resources before and after interruption.

## Additional Information and Declarations

### Data Availability

The following information was supplied regarding data availability: The raw data is available in the Supplementary File.
